# High mobility group box 1 potentiates the pro-inflammatory effects of interleukin-1β in osteoarthritic synoviocytes

**DOI:** 10.1186/ar3124

**Published:** 2010-08-27

**Authors:** Isabel García-Arnandis, Maria Isabel Guillén, Francisco Gomar, Jean-Pierre Pelletier, Johanne Martel-Pelletier, Maria José Alcaraz

**Affiliations:** 1 Department of Pharmacology, University of Valencia, Av.Vicent Andrés Estellés s/n, Burjasot, 46100 Valencia, Spain; 2 Department of Chemistry, Biochemistry and Molecular Biology, Cardenal Herrera-CEU University, Av. Seminario s/n, Moncada, 46113 Valencia, Spain; 3 Department of Surgery, School of Medicine, University of Valencia, Av. Blasco Ibáñez 15, Valencia, 46010 Valencia, Spain; 4 Osteoarthritis Research Unit, University of Montreal Hospital Research Centre (CRCHUM), Notre-Dame Hospital, Montreal, 1560 Rue Sherbrooke East, Montreal, Quebec H2L 4M1, Canada

## Abstract

**Introduction:**

High mobility group box 1 (HMGB1) is released by necrotic cells or secreted in response to inflammatory stimuli. Extracellular HMGB1 may act as a pro-inflammatory cytokine in rheumatoid arthritis. We have recently reported that HMGB1 is released by osteoarthritic synoviocytes after activation with interleukin-1beta (IL-1β) The present study investigated the role of HMGB1 in synovial inflammation in osteoarthritis (OA).

**Methods:**

HMGB1 was determined in human synovium using immunohistochemistry, comparing normal to OA. OA synoviocytes were incubated with HMGB1 at 15 or 25 ng/ml in the absence or presence of IL-1β (10 ng/ml). Gene expression was analyzed by quantitative PCR and protein expression by Western Blot and ELISA. Matrix metalloproteinase (MMP) activity was studied by fluorometric procedures and nuclear factor (NF)-κB activation by transient transfection with a NF-κB-luciferase plasmid.

**Results:**

In the normal synovium, HMGB1 was found in the synovial lining cells, sublining cells, and in the vascular wall cells. The distribution of HMGB1 in OA synovium was similar but the number of HMGB1 positive cells was higher and HMGB1 was also present in infiltrated cells. In normal synovial membrane cells, HMGB1 was found mostly in the nuclei, whereas in OA, HMGB1 was generally found mostly in the cytoplasm. In OA synoviocytes, HMGB1 alone at concentrations of 15 or 25 ng/ml did not affect the production of IL-6, IL-8, CCL2, CCL20, MMP-1 or MMP-3, but in the presence of IL-1β, a significant potentiation of protein and mRNA expression, as well as MMP activity was observed. HMGB1 also enhanced the phosphorylated ERK1/2 and p38 levels, with a lower effect on phosphorylated Akt. In contrast, JNK1/2 phosphorylation was not affected. In addition, HMGB1 at 25 ng/ml significantly potentiated NF-κB activation in the presence of IL-1β.

**Conclusions:**

Our results indicate that HMGB1 is overexpressed in OA synovium and mostly present in extracellular form. In OA synoviocytes, HMGB1 cooperates with IL-1β to amplify the inflammatory response leading to the production of a number of cytokines, chemokines and MMPs. Our data support a pro-inflammatory role for this protein contributing to synovitis and articular destruction in OA.

## Introduction

The nuclear DNA-binding protein high mobility group box 1 (HMGB1) can be passively released by necrotic cells or secreted by macrophages and other myeloid cells in response to inflammatory stimuli as part of the inflammatory response to infection or injury (reviewed in [1]). It is known that pro-inflammatory cytokines such as IL-1β or TNFα stimulate HMGB1 translocation into the cytoplasm and release in different cell types, although TNFα is not the main inducer of extracellular HMGB1 during synovitis in rheumatoid arthritis patients [2]. Oxidative stress has also been shown to induce HMGB1 release potentially through a mitogen-activated protein kinase (MAPK) and chromosome region maintenance mechanism [3].

There is abundant evidence that HMGB1 induces cell proliferation, migration and differentiation [4-6]. Binding of HMGB1 to bacterial products might create complexes inducing innate immune responses and production of inflammatory mediators. Extracellular HMGB1 interacts with receptor for advanced glycation end products (RAGE) and the toll-like receptors (TLR) including TLR-2 and TLR-4 [1] leading to the activation of monocytes, macrophages and dendritic cells. In addition, the interaction of HMGB1 with phosphatidylserine on the cell surface inhibits the phagocytosis of apoptotic neutrophils by macrophages [7], which may retard the resolution of inflammation. Nevertheless, recent studies suggest that HMGB1 alone demonstrates minor pro-inflammatory activity, which is potentiated through binding to IL-1β and other inflammatory mediators [8].

HMGB1 plays a role as a pro-inflammatory cytokine in rheumatoid arthritis and animal models of this disease. Hence, HMGB1 is overexpressed in synovial tissue of rheumatoid arthritis patients and its extracellular form has been related to the progression of arthritis in animal models [9]. Moreover, RAGE activation by HMGB1 results in increased invasiveness of fibroblast-like synoviocytes from rheumatoid arthritis patients [10].

In human articular cartilage, HMGB1 may participate in endochondral ossification during osteogenesis [11] and recently, the related protein HMGB2 has been involved in ageing and osteoarthritis (OA) [12]. Studies show that HMGB1 [13] and its receptor RAGE [14] are expressed in OA cartilage. It is also known that stimulation of OA chondrocytes with HMGB1 results in phosphorylation of extracellular signal-regulated kinase (ERK) and nuclear factor-κB (NF-κB), and matrix metalloproteinase (MMP) expression [14]. In addition, activation of RAGE by advanced glycation end products (AGEs) in OA chondrocytes and synoviocytes leads to increased catabolic activity and cartilage degeneration [15]. Nevertheless, the pro-inflammatory activity of HMGB1 in synoviocytes and its participation in synovitis during OA remain to be determined. We have recently reported that HMGB1 is released by OA synoviocytes after activation with IL-1β [16], suggesting the participation of HMGB1 in the inflammatory response induced by this cytokine. In the present study, we further investigated the role of HMGB1 in OA synovial inflammation.

## Materials and methods

### Specimen selection

Human OA knee synovial membranes were obtained from patients (12 female, 3 male, aged 69 ± 1 years, mean ± standard error of the mean (SEM)) undergoing total knee arthroplasty. All patients fulfilled the American College of Rheumatology criteria for OA of the knee [17]. Normal knees (2 female, 1 male, aged 72 ± 1.5 years) were obtained within 12 hours of death; the tissues were examined macroscopically and microscopically to ensure that only normal tissue was used. This study was approved by the Institutional Ethical Committee and is in compliance with all ethical standards and patients' consent according to the Declaration of Helsinki.

### Immunohistochemistry of HMGB1 in human synovium

HMGB1 was determined in the human synovium using immunohistochemistry, comparing normal with OA. Tissues were processed following two methodologies. Firstly, we used an HMGB1 antibody that was revealed with 3,3'-diaminobenzidine (DAB) for reading with light microscope, and secondly a fluorescent HMGB1 antibody for fluorescence microscopy analysis. In brief, the specimens were dissected and fixed in TissuFix #2 (Chaptec, Montreal, QC, Canada) and processed directly after acquisition from the donor for immunohistochemistry (basal synthesis), as previously described [18]. Sections of 5 μm of paraffin-embedded specimens were deparaffinized in toluene and rehydrated in a graded series of ethanol. The synovium was treated with Triton X-100 (0.3%) for 20 minutes at room temperature. Slides were washed in PBS followed by 3% hydrogen peroxide/methanol for 15 minutes. They were further incubated for 45 minutes with 1.5% normal serum (Vector Laboratories, Burlingame, CA, USA) and overlaid with the rabbit anti-human HMGB1 antibody (1:50 dilution; Abcam, Cambridge, MA, USA) for 18 hours at 4°C in a humidified chamber. The second antibody was a goat anti-rabbit immunoglobulin (Vector Laboratories, Burlingame, CA, USA) in which slides were incubated for 45 minutes. For reading with a light microscope, slides were stained using the avidin-biotin complex method (Vectastain ABC kit, Vector Laboratories, Burlingame, CA, USA). The colour was developed with DAB (DAKO Diagnostics Canada Inc., Mississauga, ON, Canada) without nickel chloride. The slides were counter-stained with Mayer's hematoxylin. Each section was examined under a light microscope (Leitz Orthoplan; Leica Inc., St-Laurent, QC, Canada) and photographed with a Retega OEM *Fast *camera (QImaging, Surrey, BC, Canada). For detection with fluorescence of Alexa 488 fluorochrome, slides were stained with TSA™ #22 (Invitrogen, Burlington, ON, Canada) and mounted with Vectashield (Vector Laboratories, Burlingame, CA, USA) containing 4',6-diamidino-2-phenylindole (DAPI) in order to stain the nucleus. The slides were examined under a fluorescence microscope and photographed by a CoolSNAP cf Photometrics camera (Roper Scientific, Rochester, NY, USA). Controls were performed to determine the specificity of staining: i) substitution of the primary antibodies with non-immune isotype rabbit IgG (used at the same concentration as the primary antibody); ii) immunoadsorbed with 20-fold excess of the peptide HMGB1; and iii) omission of primary antibody. Controls showed only background staining.

The presence of the antigen in the synovium was quantified by determining the number of cells that stained positive with the distinction between nuclear and extra-nuclear (cytoplasmic and cell vicinity) staining. Each synovial membrane was divided into three microscopic fields (40×; Leitz Orthoplan) and the total number of cells and those staining positive for the specific antigen were determined in the lining cells for both normal and OA, and in the infiltrates for OA specimens. The final results were expressed as the percentage of cells staining positive for the antigen with the maximum score being 100%.

Moreover, the synovial lining cell hyperplasia was graded on a scale of 0 to 2 as previously described [19], where 0 = 1 to 2 layers of cells, 1 = 3 to 5 layers, and 2 = 6 or more layers. The layers were counted from the surface of the membrane to the subsynovial tissue. Each synovium was graded into three microscopic fields (40×; Leitz Orthoplan) and the score averaged.

### Confocal microscopy

Discrimination of the HMGB1 location within the cells was confirmed using immunofluorescence followed by confocal microscopy. In brief, sections of 10 μm of paraffin-embedded specimens were treated as above. Confocal acquisitions were carried out with a Leica TCS SP5 broadband confocal microscope (St-Laurent, QC, Canada, which uses confocal point-scanning for optical sectioning. In addition, it has a spectral imaging detector allowing tunable emission bands and dye separation. The system is equipped with the AOBS (Acousto-Optical Beam Splitter) for optical beam splitting. It has three lasers: Ar 458/488/514, DPSS 561 and HeNe 633, 543, 594.

### Cell culture and treatments

Synovial specimens were finely minced and isolated by enzymatic digestion with collagenase type 1A (Sigma Aldrich, St Louis, MO, USA) in DMEM/HAM F12 (Sigma-Aldrich, St Louis, MO, USA) containing penicillin (100 U/ml) and streptomycin (100 μg/ml) at 37°C in 5% carbon dioxide atmosphere for 16 hours. The digested tissue was filtered through a 70 mm nylon mesh, washed and centrifuged. Cell viability was greater than 95% according to the Trypan blue exclusion test. Collected cells were resuspended in DMEM/HAM F12 (Sigma-Aldrich, St Louis, MO, USA) containing penicillin (100 U/ml) and streptomycin (100 μg/ml) supplemented with 10% fetal bovine serum (Sigma-Aldrich, St Louis, MO, USA) and cultured at 37°C in 5% carbon dioxide atmosphere until third passage (95% fibroblasts, detected by immunocytochemistry with anti-collagen I antibody (Chemicon, Millipore Iberica, Madrid, Spain)). Synoviocytes were allowed to grow to nearly confluence and incubated with HMGB1 (HMGBiotech, Milano, Italy) at 15 or 25 ng/ml and IL-1β (10 ng/ml, Peprotech EC Ltd, London, UK) or culture medium. Viability studies were performed for all the experimental conditions. None of the treatments significantly affected cell viability which was more than 90% as tested by Trypan blue exclusion. The possibility of endotoxin contamination of HMGB1 was excluded after performing the Limulus test using the commercial kit from Sigma-Aldrich (St Louis, MO, USA; data not shown).

### Western blot analysis

After stimulation for five minutes with IL-1β (10 ng/ml), HMGB1 at 15 and 25 ng/ml or IL-1β+HMGB1, synoviocytes were lysed in 100 μl of buffer (1% Triton X-100, 1% deoxycholic acid, 20 mM NaCl and 25 mM Tris, pH 7.4) and centrifuged at 4°C for 15 minutes at 10,000*g*. Proteins (25 μg) in cell lysates were separated by 12.5% SDS-PAGE and transferred onto polyvinylidene difluoride membranes. Membranes were blocked with 3% bovine serum albumin and incubated with specific antibodies against phosphorylated ERK (Cell Signaling Technology, Inc., Beverly, MA, USA, dilution 1:800), phosphorylated or total Akt and ERK (Cell Signaling Technology, Inc., Beverly, MA, USA; dilution 1:500), and phosphorylated or total c-Jun N-terminal kinase (JNK; Cell Signaling Technology, Inc., Beverly, MA, USA; dilution 1:250) and p38 (Promega Corporation, Madison, WI, USA; dilution 1:250) overnight at 4°C. Finally, membranes were incubated with peroxidase-conjugated goat anti-rabbit IgG (Dako, Copenhagen, Denmark) and the immunoreactive bands were visualized by enhanced chemiluminescence (GE Healthcare, city, Barcelona, Spain) using the AutoChemi image analyzer (UVP Inc., Upland, CA, USA).

### Determination of MMP activity

Cells were stimulated with IL-1β (10 ng/ml), HMGB1 at 15 and 25 ng/ml or IL-1β+HMGB1 for 24 hours and supernatants were harvested, centrifuged and incubated with p-aminophenyl mercuric acetate for six hours at 37°C to activate MMPs. Aliquots of supernatants were then transferred to a 96-well plate and after addition of the 5-FAM peptide substrate (AnaSpec Inc., San Jose, CA, USA), fluorescence was measured for different times at 490 nm (excitation)/520 nm (emission) in a Victor3 microplate reader (PerkinElmer España, Madrid, Spain).

### Enzyme-linked immunosorbent assay

Synoviocytes were stimulated with IL-1β (10 ng/ml) for 24 hours, in the presence or absence of HMGB1 at 15 and 25 ng/ml. Supernatants were harvested, centrifuged and frozen at -80°C until analysis. IL-6, IL-8 and chemokine (C-C motif) ligand 2 (CCL2) levels were determined by specific ELISA from eBioscience (San Diego, CA, USA) with sensitivity of 2, 4 and 7 pg/ml, respectively. CCL20 was determined with a specific ELISA from Raybiotech Inc. (Norcross, GA, USA) with sensitivity of 1.5 pg/ml. MMP-1 and MMP-13 protein was quantified in supernatants by using specific ELISAs from AnaSpec (sensitivity of 8 and 6 pg/ml, respectively), and MMP-3 with the ELISA from Raybiotech Inc. (sensitivity of 0.3 ng/ml).

### Real-time PCR

Following incubation for 24 hours, total RNA was extracted using the TriPure reagent (Roche Applied Science, Barcelona, Spain) according to the manufacturer's instructions. Reverse transcription was accomplished on 1 μg of total RNA using random primers (TaqMan reverse transcription reagents, Applied Biosystems, Spain, Madrid). PCR assays were performed in duplicate on an iCycler Real-Time PCR Detection System using SYBR Green PCR Master Mix (Bio-Rad Laboratories, Richmond, CA, USA) [20]. Sequences of primers used have been reported previously [21-24]. For each sample, differences in threshold cycle (DC_t_) values were calculated by correcting the C_t _of the gene of interest to the C_t _of the reference gene glyceraldehide-3-phosphate dehydrogenase. Relative gene expression was expressed as DDCt with respect to nonstimulated cells.

### Activation of NF-κB

Cells were seeded into six-well plates and grown to 50 to 60% confluence. Transient transfection was performed for 45 minutes with 2 μg of the reporter construct NF-κB-luc (Stratagene, La Jolla, CA, USA) and 1 μg of the internal control pRL-TK (Promega Corporation, Madison, WI, USA) by the Magnetofection™ system (OZ Biosciences, Marseille, France) according to the manufacturer's recommendations. The medium was then replaced and cells were treated for 24 hours with HMGB1 at 25 ng/ml in the absence or presence of IL-1β (10 ng/ml). After lysis and centrifugation, aliquots of supernatants were used to assay firefly and *Renilla *luciferase activity using the Dual-Luciferase Reporter Assay System kit (Promega Corporation, Madison, WI, USA). Luminescence was measured in a Microbeta counter (Wallac, Turku, Finland) and firefly luciferase activity was normalized to *Renilla *luciferase activity.

### Statistical analysis

Results are presented as mean ± SEM. Statistical analyses were performed using one-way analysis of variance followed by Dunnett's *t*-test for multiple comparisons and two-tailed unpaired Student's *t*-test for dual comparisons.

## Results

### HMGB1 expression in human synovium

**Figure 1:**
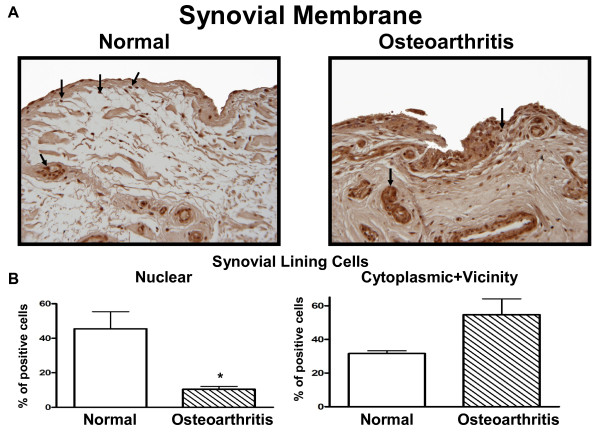
**Expression of HMGB1 protein in human synovium**. **(a) **Representative immunohistochemical sections of normal (*n *= 3) and osteoarthritic (*n *= 3) human synovium with high mobility group box 1 (HMGB1) revealed in 3,3'-diaminobenzidine (DAB). Arrows indicate HMGB1 positive cells. Original magnification × 100. **(b) **Histograms of normal and osteoarthritic positive cells (%) in the synovial lining cell nucleus and cytoplasm and vicinity. Data are expressed as mean ± standard error of the mean (*n *= 3). *P < 0.05 with respect to normal.

In the normal and OA synovium, HMGB1 was found in the cells of the synovial lining, sublining cells, and in the cells of the vascular wall (i.e. cells around the blood vessels) (Figure 1a). We further performed an analysis of the percentage of positive synoviocytes in the nuclear and extra-nuclear (cytoplasmic and cell vicinity) compartments of the cells comparing normal (*n *= 3) with OA (*n *= 3). As illustrated in Figure 1b, a statistically significant decrease in HMGB1 was found in OA cell nuclei when compared with normal (*P *< 0.03), and, although there was more positive staining in the extra-nuclear (cytoplasm and in the cell vicinity) compartment, this did not quite reach statistical significance (*P *< 0.07). In OA synovium, the percentage of infiltrate cells staining positive in the nuclei (23.6 ± 3.9%) was significantly less than in the extra-nuclear compartment (52.4 ± 4.1%; *P *< 0.007). Moreover, comparison of the synovial lining layers showed, as expected, more cell layers in the OA (score:1.3 ± 0.2) than in the normal (0.6 ± 0.2) synovial membrane.

**Figure 2:**
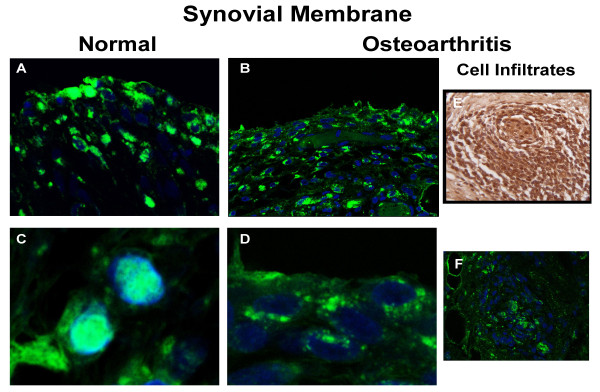
**Confocal analysis of HMGB1 expression in human synovium**. Representative immunohistochemical sections of human **(a, c) **normal (*n *= 3) and **(b, d) **osteoarthritic (*n *= 3) synovium with high mobility group box 1 (HMGB1) revealed in fluorescence and read with confocal microscopy. **(e) **represents immunohistochemistry revealed in 3,3'-diaminobenzidine (DAB) of human osteoarthritic synovium infiltrates, and **(f) **is as (e) but revealed with the fluorescent antibody. The colour green represents HMGB1 and blue the nucleus. Original magnification × 100. (c) and (d) were amplified by the image processor software.

Experiments performed with the fluorescent antibody (Figure 2) confirmed that in normal synovial membrane cells, HMGB1 (green) was found mostly in the nuclei with some cells showing cytoplasm staining (Figures 2a to 2c). In OA (Figures 2b to 2d), however, HMGB1 was generally found mostly in the cytoplasm. For cell infiltrates (Figures 2e to 2f), the pattern was as for the OA lining cells, in which HMGB1 was found mostly in the cytoplasm.

### Effect of HMGB1 on cytokine and chemokine production induced by IL-1β in OA synoviocytes

**Figure 3:**
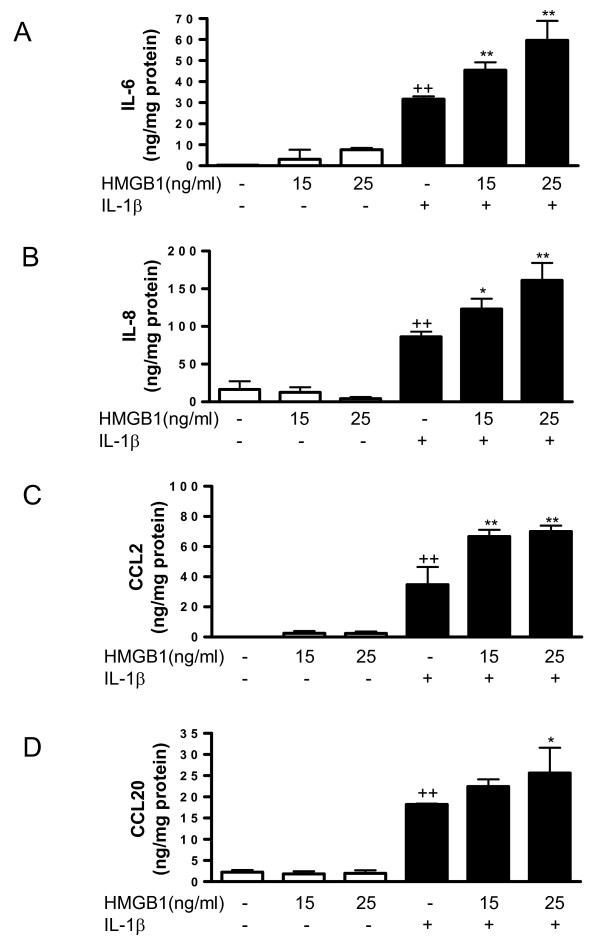
**Effect of HMGB1 and IL-1β on the levels of cytokine and chemokine released into the medium by osteoarthritic synoviocytes**. **(a) **IL-6, **(b) **IL-8, **(c) **CCL2 and **(d) **CCL20 protein levels. Cells were stimulated with IL-1b (10 ng/ml) for 24 hours in the presence or absence of high mobility group box 1 (HMGB1) at 15 and 25 ng/ml. Protein levels were determined in supernatants by ELISA. Data are expressed as mean ± standard error of the mean. Duplicate samples from six patients were used. ++*P *< 0.01 with respect to nonstimulated cells. *P < 0.05, **P < 0.01 with respect to IL-1β.

**Figure 4:**
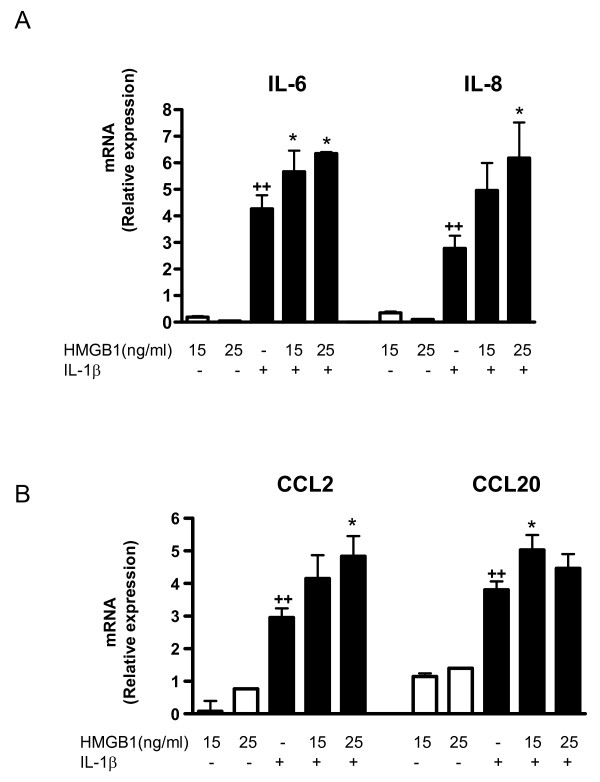
**Effect of HMGB1 and IL-1β on cytokine and chemokine mRNA levels in OA synoviocytes**. **(a) **IL-6, IL-8, and **(b) **CCL2 and CCL20 mRNA relative expression. Cells were stimulated with IL-1b (10 ng/ml) for 24 hours in the presence or absence of high mobility group box 1 (HMGB1) at 15 and 25 ng/ml. mRNA expression was determined by real-time PCR. Data are expressed as mean ± standard error of the mean. Duplicate samples from four patients were used. ++*P *< 0.01 with respect to nonstimulated cells. *P < 0.05 with respect to IL-1β.

To determine whether extracellular HMGB1 can modulate cytokine and chemokine production in human OA synoviocytes, cells were incubated with HMGB1 in the presence or absence of IL-1β (10 ng/ml). This pro-inflammatory cytokine is present in OA synovial fluid and cartilage and participates in joint degradation [25,26]. As expected, stimulation with IL-1β resulted in the enhanced production of pro-inflammatory cytokines and chemokines. As shown in Figure (3a to 3d), although HMGB1 alone at concentrations of 15 or 25 ng/ml did not affect the production of IL-6, IL-8, CCL2 or CCL20, a significant potentiation of these pro-inflammatory mediators was observed in the presence of IL-1β. These effects of HMGB1 were confirmed at the mRNA level, with an enhancement of IL-6, IL-8, CCL2 and CCL20 mRNA expression in cells stimulated with IL-1β (Figures 4a and 4b).

### Effect of HMGB1 on MMPs induced by IL-1β in OA synoviocytes

**Figure 5:**
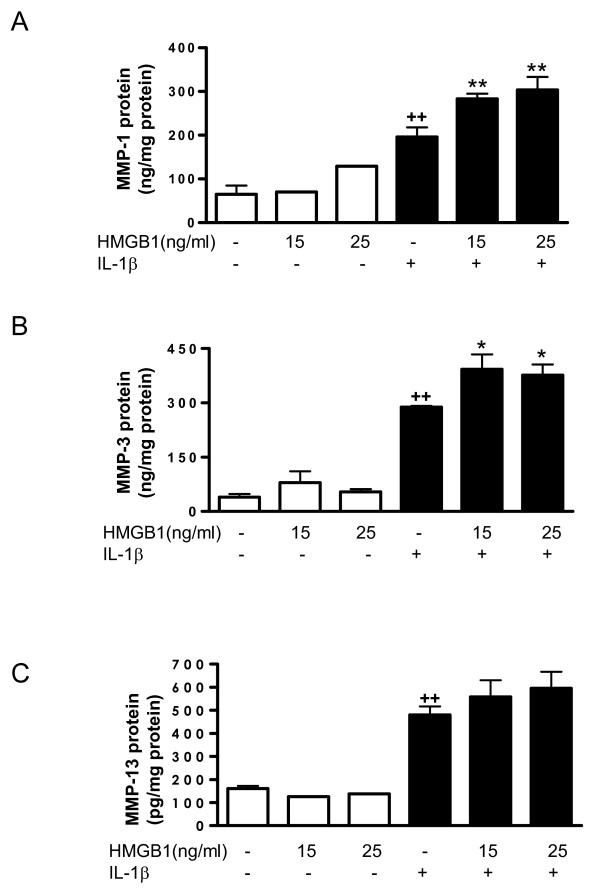
**Effect of HMGB1 and IL-1β on the levels of MMP released into the medium by osteoarthritic synoviocytes**. **(a) **Matrix metalloproteinase (MMP)-1, **(b) **MMP-3 and **(c) **MMP-13 protein levels in the medium. Cells were stimulated with IL-1b (10 ng/ml) for 24 hours in the presence or absence of high mobility group box 1 (HMGB1) at 15 and 25 ng/ml. MMP protein was measured by ELISA in supernatants. Data are expressed as mean ± standard error of the mean. Duplicate samples from six to eight patients were used. ++*P *< 0.01 with respect to nonstimulated cells. *P < 0.05, **P < 0.01 with respect to IL-1β.

**Figure 6:**
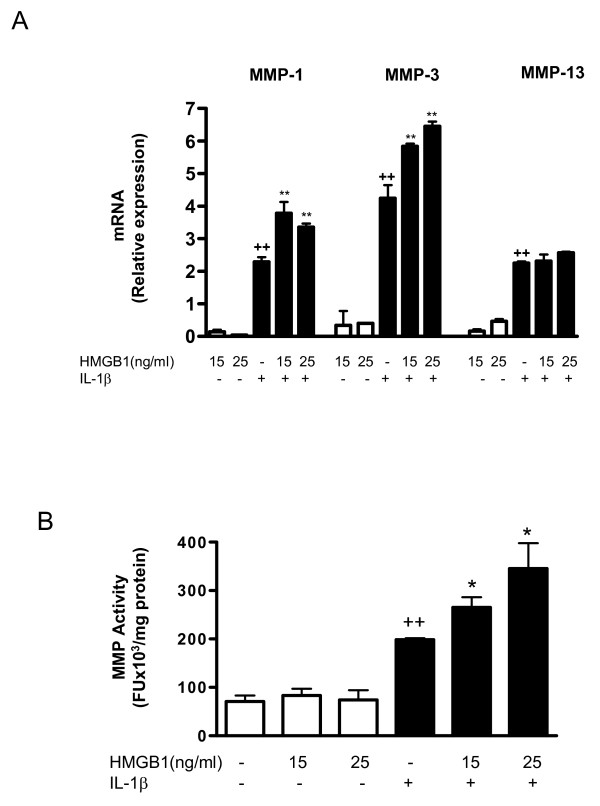
**Effect of HMGB1 and IL-1β on MMP mRNA levels in osteoarthritic synoviocytes and MMP activity released into the medium**. **(a) **Matrix metalloproteinase (MMP)-1, MMP-3 and MMP-13 mRNA levels and **(b) **MMP activity. Cells were stimulated with IL-1b (10 ng/ml) for 24 hours in the presence or absence of high mobility group box 1 (HMGB1) at 15 and 25 ng/m. mRNA was determined by real-time PCR and MMP activity was measured in supernatants by a fluorometric procedure, as indicated in Materials and Methods. Data are expressed as mean ± standard error of the mean. Duplicate samples from four patients were used. ++*P *< 0.01 with respect to nonstimulated cells. *P < 0.05, **P < 0.01 with respect to IL-1β.

Cell activation by IL-1β (10 ng/ml) potently induced MMP gene expression as well as MMP protein and activity. mRNA expression was measured by real-time PCR. In cell supernatants, protein levels were measured by ELISA and MMP activity by a fluorometric procedure. As shown in Figure (5a to 5c), HMGB1 alone did not induce significant changes in MMP-1, MMP-3 or MMP-13 protein in cell supernatants, but it potentiated the stimulating effect of IL-1β for MMP-1 and MMP-3. In addition, HMGB1 significantly increased MMP-1 and MMP-3 mRNA expression in the presence of IL-1β (Figure 6a). These effects were reflected in the levels of MMP activity released into the medium, which were significantly increased by HMGB1 after IL-1β stimulation (Figure 6b).

### Effect of HMGB1 on Akt and MAPK phosphorylation induced by IL-1β in OA synoviocytes

**Figure 7:**
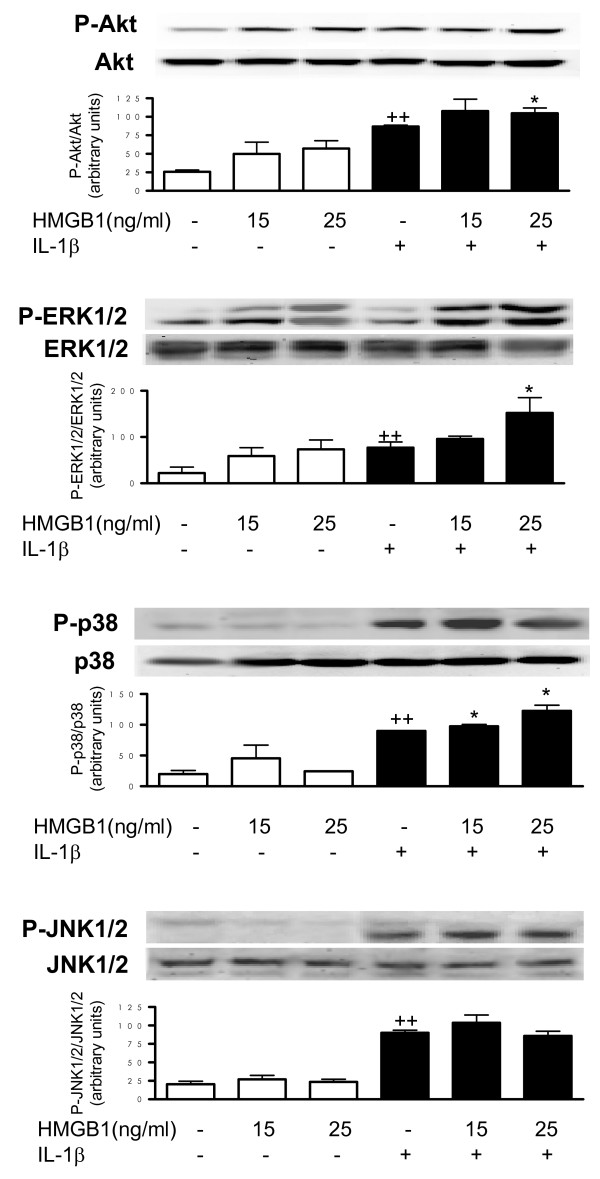
**Effect of HMGB1 and IL-1β on Akt and MAPK phosphorylation**. Cells were stimulated with IL-1b (10 ng/ml) for five minutes in the presence or absence of high mobility group box 1 (HMGB1) at 15 and 25 ng/ml. Protein level was determined in cell lysates by western blotting by using specific antibodies against phosphorylated or total proteins. Relative expression of phosphorylated and total protein bands was calculated after densitometric analysis. Data are expressed as mean ± standard error of the mean (samples from three patients). ++*P *< 0.01 with respect to nonstimulated cells. *P < 0.05, with respect to IL-1β. ERK, extracellular signal-regulated kinase; JNK, c-Jun N-terminal kinase; MAPK, mitogen-activated protein kinase.

To determine the possible mechanism of action of HMGB1, we further examined whether this protein acts on Akt and MAPK activation. The time of stimulation for a high phosphorylation response was chosen from previous experiments [see Additional file 1]. As shown in Figure 7, HMGB1 at both concentrations studied (15 and 25 ng/ml) increased the phosphorylated Akt and ERK1/2 levels. In the presence of IL-1β, HMGB1 enhanced the phosphorylated ERK1/2 and p38 levels, with a lower effect on phosphorylated Akt. In contrast, JNK1/2 phosphorylation was not affected by HMGB1 either in the presence or absence of IL-1β stimulation.

### Effect of HMGB1 on NF-κB activation induced by IL-1β in OA synoviocytes

**Figure 8:**
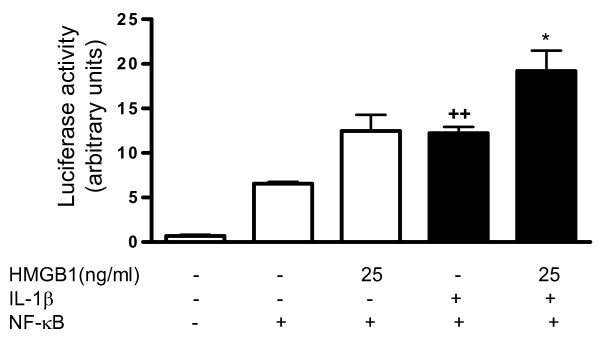
**Effect of HMGB1 and IL-1β on NF-κB activation**. Transient transfection was performed with the reporter construct nuclear factor (NF)-κB-luc and the internal control pRL-TK, as indicated in Materials and Methods. Cells were treated for 24 hours with high mobility group box 1 (HMGB1) at 25 ng/ml in the absence or presence of IL-1β (10 ng/ml). Firefly luciferase activity was normalized to *Renilla *luciferase activity. Data are expressed as mean ± standard error of the mean. Duplicate samples from six patients were used. ++*P *< 0.01 with respect to nonstimulated cells; *P < 0.05 with respect to IL-1β.

NF-κB is a main regulator of pro-inflammatory and degradative genes in the joint. As IL-1β [27] and HMGB1 [28] cell stimulation results in the activation of this pathway in different cell types, we studied the possible participation of this mechanism in the pro-inflammatory effects of HMGB1 in OA synoviocytes. HMGB1 treatment in nonstimulated cells resulted in increased transcriptional activity, although it did not reach statistical significance. Of note, HMGB1 at 25 ng/ml significantly potentiated NF-κB activation in the presence of IL-1β (Figure 8).

## Discussion

Synovial inflammation has been demonstrated in tissue samples of OA patients and may be related to disease progression [17,29,30]. Several lines of evidence support the involvement of synoviocytes in OA cartilage degradation through the production of inflammatory and catabolic mediators [31]. In this regard, pro-inflammatory cytokines such as IL-1β play a role in driving synovitis during OA and influencing the production of cytokines and MMPs [32]. With respect to the biological role of HMGB1, it is essential to understand the mechanisms involved in the regulation of the inflammatory response. HMGB1 appears to act as a pro-inflammatory cytokine in mononuclear cells through the release of TNFα, IL-1β, IL-6, IL-8, macrophage inflammatory protein-1 and nitric oxide [4,33]. In addition, HMGB1 stimulates the motility of a wide range of cells and thus elicits the migration to the site of tissue damage, a process dependent on the activation of ERK, NF-κB [34] and Src [35] pathways.

Our data first showed that less HMGB1 is observed in the nuclei of OA synoviocytes, and although a higher level is found in the cytoplasm and vicinity in these cells, this did not quite reach statistical significance. This lack of significance could be related to the low number of specimens analysed; however, as HMGB1 is released extra-cellularly, our evaluation could be falsely lower. Yet, as there were more cell layers in OA compared with normal, in addition to the presence of cell infiltrates in OA showing an elevated level of HMGB1 in the extra-nuclear compartment compared with the nuclear, this strongly suggests that more HMGB1 is released from OA synovial cells than from normal, supporting the previous data in which OA cells released HMGB1 upon activation by IL-1β [16]. We thus hypothesized that HMGB1 may play a role in modulating the inflammatory process in synovium during OA. Our results indicate that HMGB1 cooperates with IL-1β to amplify the inflammatory response leading to the production of a number of cytokines, chemokines and MMPs in OA synoviocytes. Data showed that HMGB1 plus IL-1β synergistically enhanced IL-6 production, which is in line with a recent report on the stimulation of IL-6 release by preformed complexes of HMGB1 (100 ng/ml) and IL-1β at a low concentration unable to stimulate synoviocytes (0.05 ng/ml). Interestingly, the response of synovial fibroblasts from OA or rheumatoid arthritis patients was similar [36].

Chemokines such as IL-8, CCL2 and CCL20 are produced by OA synoviocytes, but to a lower extent than rheumatoid arthritis cells. These mediators have the ability to attract inflammatory cells and regulate gene transcription and cell proliferation [37]. The up-regulation of chemokines upon IL-1β synoviocyte stimulation promotes inflammation and cartilage degradation through the activation of MMPs and other degradative enzymes [38]. CCL2 and CCL20 are chemokines implicated in rheumatoid arthritis synovitis [39-41] and are produced by OA synovium in the presence of pro-inflammatory cytokines. In particular, IL-1β has been shown to be a more potent inducer of CCL20 than TNFα or IL-17 [42]. Our data revealed that HMGB1 acted on OA synoviocytes *in vitro *to enhance the production of IL-8, CCL2 and CCL20. The best studied CXC chemokine is IL-8, which is produced by fibroblasts and macrophages present in synovial tissues [43]. Induction of synovial macrophage and fibroblast chemotaxis has been demonstrated for CCL2 [40,44], whereas CCL20 induces monocyte and memory lymphocyte chemotaxis from peripheral blood to the rheumatoid joint [45]. Therefore, the enhanced production of these mediators in the presence of HMGB1 supports a role for this protein in the amplification of the inflammatory response induced by IL-1β in OA synoviocytes.

MMPs play an important role in articular tissue degradation in OA. The present study demonstrated the potentiating effect of HMGB1 on IL-1β induction of MMP-1 and MMP-3 in human OA synoviocytes. As MMP-1 degrades collagens in the extracellular matrix [46-48] and MMP-3 activity leads to activation of collagenases [49], our results suggest the amplification of catabolic responses by HMGB1 during joint inflammation.

Several studies indicate that spontaneous or stimulated production of many inflammatory and degradative mediators by OA synoviocytes are related to NF-κB activation [50,51]. In particular, transcription of IL-6 [52], IL-8 [53], CCL2, CCL20 [41,54] and MMPs [55,56] is NF-κB dependent. Our data show that IL-1β plus HMGB1 synergistically increased the transcriptional activity of NF-κB, leading to an enhanced production of inflammatory and catabolic mediators.

MAPK activity regulates the activation of transcription factors relevant in inflammatory responses [57]. We have shown that HMGB1 enhances p38 phosphorylation, which participates in IL-6 and IL-8 transcription in human fibroblast-like synoviocytes [58,59]. As the production of MMP-1 and MMP-3 upon stimulation of synoviocytes with IL-1β depends on ERK activation [60], we investigated the effects of HMGB1 on ERK phosphorylation. Our results indicate that HMGB1 potentiates the effects of IL-1β on ERK phosphorylation, which may play a role in the up-regulation of MMP-1 and MMP-3 by HMGB1. In contrast, we did not observe any modification of JNK1/2, which phosphorylates c-Jun and regulates the transcription factor activating protein-1 [61] as well as erythroblastosis 26 transcription factors [62]. Interestingly, HMGB1 potentiates Akt phosphorylation by IL-1β, a pathway involved in cell survival and proliferation of fibroblasts in rheumatoid arthritis synovium [63]. In addition, Akt activation may play a role in human cartilage breakdown, because it has been implicated in MMP-13 and aggrecanase-1 expression induced by oncostatin M [64] and the synergistic induction of MMP-1 and MMP-13 expression after oncostatin M+IL-1β stimulation of human chondrocytes [65]. Our data would therefore suggest that the potentiation of ERK, p38 and Akt activation by HMGB1 may be a mechanism relevant for the increase in the intensity and persistence of synovitis as well as the expression of catabolic factors in OA.

## Conclusions

HMGB1 is overexpressed in synovial membranes of OA patients. The results presented here support the view that HMGB1 would act as a pro-inflammatory cytokine, which enhances the OA synovial inflammatory process. HMGB1 was found to synergize with IL-1β to induce phosphorylation of ERK1/2, p38 and Akt, as well as NF-κB activation. These effects result in the production of pro-inflammatory and catabolic mediators that would contribute to synovitis and articular destruction during OA.

## Abbreviations

AGE: advanced glycation end product; CCL: chemokine (C-C motif) ligand; DAB: 3,3'-diaminobenzidine; DAPI: 4',6-diamidino-2-phenylindole; ELISA: enzyme-linked immunosorbent assay; ERK: extracellular signal-regulated kinase; HMGB1: high mobility group box 1; IL: interleukin; JNK: c-Jun N-terminal kinase; MAPK: mitogen-activated protein kinase; MMP: matrix metalloproteinase; NF-κB: nuclear factor-κB; OA: ostearthritis; PBS: phosphate-buffered saline; PCR: polymerase chain reaction; RAGE: receptor for advanced glycation end products; SEM: standard error of the mean; TLR: toll-like receptor; TNFα: tumor necrosis factor-α.

## Competing interests

The authors declare that they have no competing interests.

## Authors' contributions

IG and MIG carried out the experimental work and the data collection and interpretation. FG participated in the design and coordination of experimental work, and acquisition of data. J-PP and JM-P participated in the study design, data collection, analysis of data and preparation of the manuscript. MJA carried out the study design, the analysis and interpretation of data and drafted the manuscript. All authors read and approved the final manuscript.

## Supplementary Material

Additional file 1 **Supplementary figure S1**. Time course of high mobility group box 1 (HMGB1) and IL-1β effects on Akt and mitogen-activated protein kinase (MAPK) phosphorylation. Click here for file
