# Supplementary material for: High mobility group box 1 potentiates the pro-inflammatory effects of interleukin-1β in osteoarthritic synoviocytes
Source: Arthritis Res Ther. 2010 Aug 27;12(4):R165. doi: 10.1186/ar3124 (PMC2945068; doi:10.1186/ar3124)
Supplement: Additional file 1 — Supplementary figure S1. Time course of high mobility group box 1 (HMGB1) and IL-1β effects on Akt and mitogen-activated protein kinase (MAPK) phosphorylation. [file ar3124-S1.PDF]

Supplementary FIG. 1

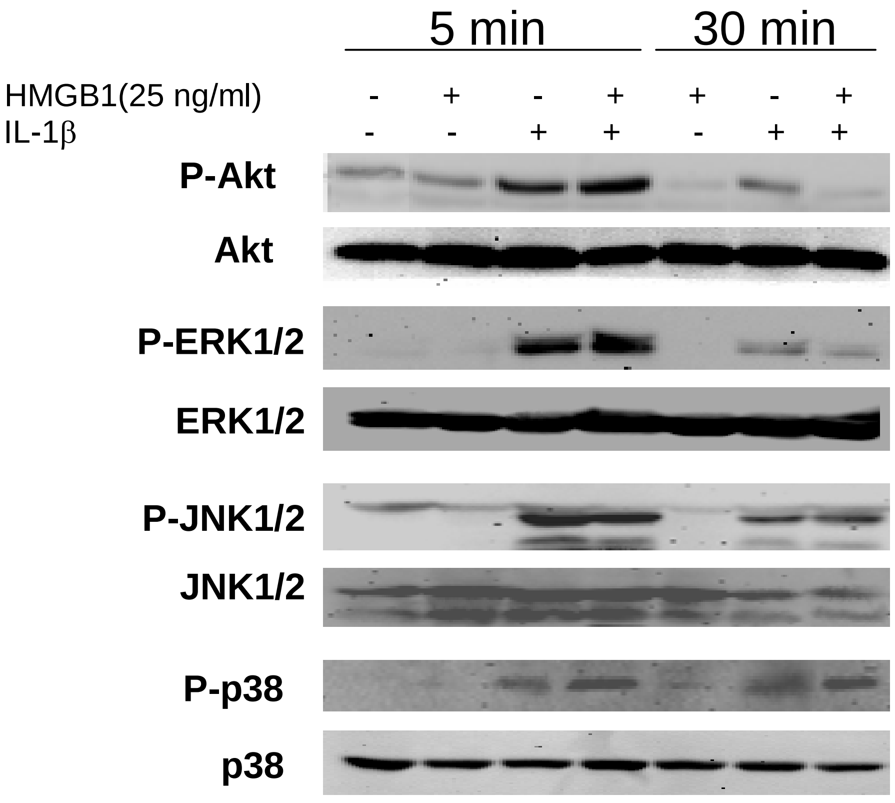

Supplementary Figure 1. Time course of HMGB1 and IL-1 β effects on Akt and MAPK phosphorylation. Cells were stimulated with IL-1β (10 ng/ml) for 5 or 30 min in the presence or absence of HMGB1 at 25 ng/ml. Protein level was determined in cell lysates by Western blotting by using specific antibodies against phosphorylated or total proteins. Representative immunoblot of samples from 2 patients.
